# Tactile input and empathy modulate the perception of ambiguous biological motion

**DOI:** 10.3389/fpsyg.2015.00161

**Published:** 2015-02-20

**Authors:** Hörmetjan Yiltiz, Lihan Chen

**Affiliations:** ^1^Department of Psychology, Peking UniversityBeijing, China; ^2^Key Laboratory of Machine Perception (Ministry of Education), Peking UniversityBeijing, China

**Keywords:** tactile, point-light walker, temporal, empathy, apparent motion, binocular rivalry

## Abstract

Evidence has shown that task-irrelevant auditory cues can bias perceptual decisions regarding directional information associated with biological motion, as indicated in perceptual tasks using point-light walkers (PLWs) (Brooks et al., 2007). In the current study, we extended the investigation of cross-modal influences to the tactile domain by asking how tactile input resolves perceptual ambiguity in visual apparent motion, and how empathy plays a role in this cross-modal interaction. In Experiment 1, we simulated the tactile feedback on the observers' fingertips when the (upright or inverted) PLWs (comprised of either all red or all green dots) were walking (leftwards or rightwards). The temporal periods between tactile events and critical visual events (the PLW's feet hitting the ground) were manipulated so that the tap could lead, synchronize, or lag the visual foot-hitting-ground event. We found that the temporal structures between tactile (feedback) and visual (hitting) events systematically biases the directional perception for upright PLWs, making either leftwards or rightwards more dominant. However, this effect was absent for inverted PLWs. In Experiment 2, we examined how empathy modulates cross-modal capture. Instead of giving tactile feedback on participants' fingertips, we gave taps on their ankles and presented the PLWs with motion directions of approaching (facing toward observer)/receding (facing away from observer) to resemble normal walking postures. With the same temporal structure, we found that individuals with higher empathy were more subject to perceptual bias in the presence of tactile feedback. Taken together, our findings showed that task-irrelevant tactile input can resolve the otherwise ambiguous perception of the direction of biological motion, and this cross-modal bias was mediated by higher level social-cognitive factors, including empathy.

## Introduction

Perceiving and recognizing biological motion patterns in a complex and cluttered environment is vital for human survival. Our understanding of the perception of biological motion has been increased by advancements in research methodology and paradigms (Cutting and Kozlowski, 1977; Cutting, 1978; Watson et al., 2004; Kim et al., 2010; van Boxtel and Lu, 2013). One development in methodology that has benefitted research in this domain is the use of point-light walkers. Johansson first introduced point-light walkers to examine how well human observers could extract motion and form information for a simulated walking person from the characteristic light dots rendering key parts of the human body (Johansson, 1973). This novel paradigm proved to be very successful and has been used extensively to investigate perceptual organization and visual attention in complex environments for more than two decades (Schmuckler and Fairhall, 2001; Servos et al., 2002; Beauchamp et al., 2003; Hirai and Hiraki, 2005; Troje et al., 2006; Brooks et al., 2007; Arrighi et al., 2009; Das et al., 2009; Hirai et al., 2009; Herrington et al., 2011; Pavlova et al., 2014). Researchers initially examined how observers could use visual cues to facilitate the detection of certain features (either static or dynamic motion information) among the given PLWs (Das et al., 2009; de Lussanet and Lappe, 2012).

Studies have also addressed how people process social information that is embedded in the PLW, such as gender (Barclay et al., 1978; Pollick et al., 2005) and emotion (Ma et al., 2006; Johnson et al., 2011; Henry et al., 2012). Perception of PLWs has been shown to be modulated by individual differences and personality traits, such as age (Norman et al., 2004), identity (Barclay et al., 1978; Cutting, 1978; Troje et al., 2005), and the self-serving bias. Regarding the self-serving bias, a recent study revealed that in perceiving the receding/approaching directional information for PLWs, observers with high social anxiety are less likely to report the PLW as approaching, compared to observers with low social anxiety. This bias might reflect an assumption that mistaking approach for withdrawal is worse than the reverse (Van de Cruys et al., 2013; Weech et al., 2014).

In naturalistic settings and daily life however, it is often the case that biological motion involves information from more than one modality. Thus, research into the role of multi-modal information in biological motion is necessary for a more comprehensive understanding of biological motion. While studies utilizing PLWs were originally confined to the visual modality, they have fortunately been extended to a multisensory context in recent years. In particular, several studies have targeted how auditory inputs resolve the otherwise ambiguous directional perception of PLWs. Brooks et al. (2007) investigated the effect of suprathreshold auditory motion on perceptions of visually-defined biological motion. Here, researchers manipulated the same (congruent) or opposite (incongruent) directions between auditory motion and visual motion, and found a direction-congruent effect between auditory events and visual PLWs. Relative to control auditory conditions, auditory motion in the same direction as the visually-defined biological motion target increased its detectability. However, it decreased detectability of the biological motion target when the directions of auditory motion and the visual PLW were incongruent (Brooks et al., 2007). In a similar vein, Kim et al. (2010) found a general improvement for the detection of a point-light *talking* face among point-light distractors, in the presence of congruent/matched auditory speech. This suggests that concomitant action-consistent sounds enhance visual sensitivity to the presence of coherent point-light displays of human movement. Thomas and Shiffrar (2010) examined further whether the visual detection sensitivity of PLWs is modulated by the meaningfulness of sounds that are concomitant with observed point-light actions. They revealed that detection sensitivity increased as a result of the veridical auditory cues (footfalls) but not as a result of pure tones. Taken together, the above studies suggest that the correspondence of auditory information to visual information, whether in lower perceptual features (direction) or higher cognitive factors (semantic relatedness), could to a large extent enhance visual sensitivity to the presence of coherent point-light displays of human movement.

The cross-modal influence of sensory inputs on perception of PLWs was driven mainly by temporal factors. For instance, performance on identifying upright PLWs was better when the visual “footsteps” were phase-locked with the auditory events. However, this advantage disappeared when the visual footsteps were out of phase with the auditory events (Saygin et al., 2008). The cross-modal influence on the temporal “capture” effect has been termed the “temporal ventriloquism effect.” In a typical dynamic ventriloquism effect, the perceived direction of the bistable visual motion (either leftwards or rightwards) is discerned by temporal alignments between distractor events (auditory events) and target (visual or tactile) events in the apparent motion (Slutsky and Recanzone, 2001; Bertelson and Aschersleben, 2003; Morein-Zamir et al., 2003; Vroomen et al., 2004; Shi et al., 2010; Chen and Vroomen, 2013). However, the distractor events provided no spatial cue (or motion direction) information and the temporal disparity between cross-modal events was beyond conscious perception (Freeman and Driver, 2008; Chen et al., 2011).

The current study aims to extend the research just discussed. Its purpose is two-fold. First, tactile events, like auditory signals, share the Gestalt principle of perceptual organization, so that paired tactile events could serve as temporal cues to influence the timing of visual/auditory events, and even cause a multisensory illusion-ventriloquism effect (Gallace and Spence, 2010, 2011). Therefore, events from a third modality, such as tactile input associated with veridical and ecologically meaningful feedback on the visual footfalls of PLWs, could affect the perception of PLWs. This would be the case as long as there was appropriate temporal alignment between the onset times of the tactile inputs and the motion simulated by the PLW. Investigation along this line has not yet been documented. Therefore, we aimed to examine how the tactile temporal perceptual grouping (with visual frames of PLWs) influences the perception of the directional information of PLWs. The effect of the cross-modal temporal capture was measured by the variation in the perceived dominant durations of PLWs in one direction.

Second, as we described previously, perception of PLWs mobilizes not only low-level visual processing, but involves high-level cognitive inputs such as the cognitive states of the observers, due to the fact that PLWs can invoke social and emotional responses (Van de Cruys et al., 2013). Social neuroscience models have assumed that people tend to use the self as a reference point to perceive the world and gain information about other people's mental states. Further, people rely mainly on their own cognitive states as a reference for empathy (Silani et al., 2013). Recent studies have also shown the neural basis for invidual differences in empathy. Somatosensory response in the primary somatosensory cortex (SI) has been associated with the empathy subscale of perspective taking (Schaefer et al., 2012). This link demonstrates that vicarious somatosensory responses for simple touch are influenced by the observer's personality traits. That is, people with higher empathic concern would be more sensitive to other individuals' suffering (Banissy and Ward, 2007). We intend to apply tactile feedback to the participants as vicarious feedback from the PLWs. This essentially requires the participants to associate the experience of the first-person (the participant) and the third person (the PLWs) when they interpret the motion state of the PLWs with (dissociated) tactile feedback. From the above reasoning, we speculate that people with higher empathy will involve themselves more in the current cross-modal interaction task (Gallese et al., 2004; Cattaneo and Rizzolatti, 2009), and would therefore show a modulation effect of empathy upon the tactile temporal capture effect. Among the many operational techniques in PLWs, binocular rivalry remains a rigorous paradigm that induces potential perceptual bistability (Watson et al., 2004). This could however, be explained by different factors, including postures and cross-modal sensory inputs (Brooks et al., 2007; Kim et al., 2010).

Using the paradigm of binocular rivalry, we conducted two experiments to test the following hypotheses: (1) Tactile events as simulations of visual footsteps could help to organize the directional information of the otherwise ambiguous/bistable apparent motion of PLWs; (2) The tactile-visual dynamic temporal capture effect of the directional perception of PLWs is constrained by higher-level social-cognitive factors, including an individual's empathy.

## Experiment 1

### Method

#### Participants

Sixteen undergraduate students (7 female) from Peking University, aged 19–23 years, with normal or corrected-to-normal vision participated in the experiment. None of them had color-blindness or partial colorblind symptoms, they reported normal hearing, and normal somatosensory sensation. The experiment was conducted on each participant individually, in a dimly lit standard experimental booth. The experiment was performed in compliance with all institutional guidelines set by the Academic Affairs Committee of the Department of Psychology at Peking University. All participants provided written informed consent according to institutional guidelines and the Declaration of Helsinki. Participants were reimbursed after the experiment.

#### Stimuli and apparatus

The raw data for composing the point-light walker's stimuli were obtained from CMU Graphics Lab Motion Capture Database (http://mocap.cs.cmu.edu). We presented two point-light walkers. Each PLW was either completely red or completely green, and was either upright or inverted. A point-light walker consisted of 13 dots, representing some of the key joints of the body, including the head, shoulders, elbows, hands, hips, knees, and feet (Ahlstrom et al., 1997). Each PLW extended approximately 6 (high) × 4 (wide) degrees of visual angle on screen, viewed from a distance of 60 cm to the eyes of the observer. The distance between the center of the two PLWS was kept at 16 cm, where the walking direction for each PLW was either leftwards or rightwards. However, the two PLWs were mirror-reflected in the stereoscope so that they converged and overlapped at the center of the screen. As a result, each eye of the observer only saw a single PLW at the corresponding side, which induces binocular rivalry (see the following procedure). The walking directions for the PLWs in each trial were randomized and counterbalanced. A full walking cycle for a PLW was 1300 ms, with 130 frames presented at a vertical refresh rate of 10 ms per frame. The visual display was a 19 inch CRT (ViewSonic) with a resolution of 1024 × 768, at a vertical refresh rate of 100 Hz, which enabled the inter-frame time interval between visual stimuli to be set at 10 ms. Red and green stimuli were equiluminant at 14.88 and 10.49 cd/m^2^ respectively, on a black screen background with a luminance of 0.17 cd/m^2^.

**Figure 1:**
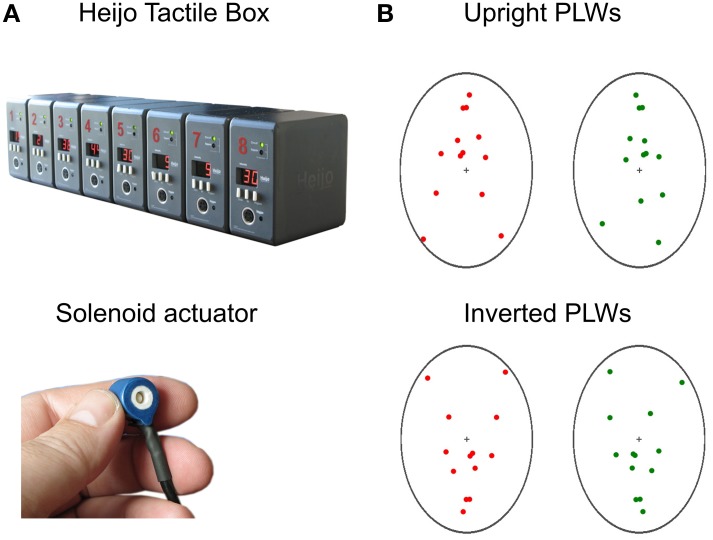
**The Heijo Tactile box and solenoid actuator (A) and the PLWs with upright and inverted postures (B)**. Here we used two channels of tactile actuators which tapped the two forefinger tips. For the PLWs in the upright condition, both red and greed point-light walkers were upright, with opposite walking direction positioned symmetrically at the left and right sides of the screen with a center to center distance of 16 cm. The background used in the experiment was black for both the upright and the inverted PLWs. However, in illustrating the PLWs here, we used a white background. When viewed through the stereoscope, the walkers overlapped, inducing binocular rivalry. A whole walking cycle lasted 1300 ms. In the inverted condition, both walkers were presented upside-down with the same inter-distance and timing parameter.

**Figure 2:**
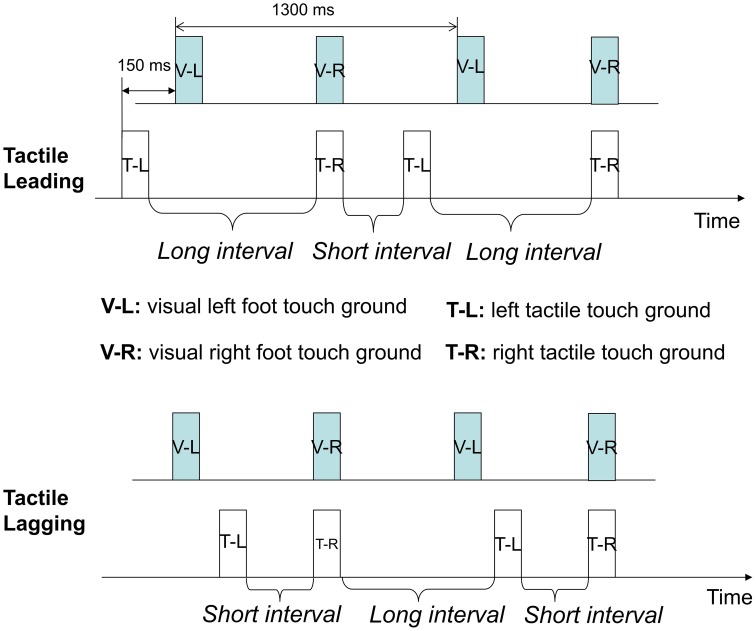
**Temporal structures of visual-tactile stimuli in PLWs**. Here, two of the eight experimental conditions of Experiment 1 are shown. The upper figure shows the “tactile leading” temporal condition, in which one tap was leading 150 ms to one visual footstep (visually touching the ground), while the other tap was synchronous with the second visual footstep. In contrast, the lower figure shows the tactile lagging condition, in which one tap was lagging 150 ms to one visual footstep while the other tap was synchronous with the onset of the second visual footstep. V, visual; T, tactile feedback (tap); L, left; R, right.

The tactile stimuli were produced using solenoid actuators with embedded cylinder metal tips, which would tap the fingertips to induce indentation taps when the solenoid coils were magnetized (Heijo Box, Heijo Research Electronics, UK, as shown in Figure 1). The maximum contact area is about 4 mm^2^ and the maximum output is 3.06 W. Two tactile stimuli, simulating one of the (randomly chosen by trial) point-light walker's footsteps touching the ground, were presented on the index fingers. The temporal structures for the tactile stimuli and visual stimuli were as follows: the first tactile stimulus for each trial (e.g., the left tactile stimulus simulating the tactile feedback of a visual left footstep) was synchronized with the corresponding visual stimulus (e.g., the left visual footstep) for the whole trial. The second tactile stimulus either *preceded* 150 ms, *synchronized*, or *lagged* 150 ms to the corresponding visual frame of the PLW's footstep hitting the ground, as shown in Figure 2. The duration for a single tap lasted 10 ms. Each initial tap was assigned to either the left forefinger tip or the right forefinger tip. The order was randomized and counterbalanced across all experimental trials, also shown in Figure 2. To give more detail, in the “tactile leading” temporal condition, one tap was leading 150 ms to one visual footstep (visually touching the ground), while the other tap was synchronous with the second visual footstep. In contrast, the lower figure showed the “tactile lagging” condition, in which one tap was lagging 150 ms to one visual footstep while the other tap was synchronous with the onset of the second visual footstep. The pairing of visual and tactile stimuli could be organized into interleaved short intervals and long intervals along the whole presentation duration (70 s) of PLWs. There were another two conditions: “synchronous” and “baseline.” In the synchronous condition, both taps were synchronous with the corresponding critical visual footsteps (hitting twice on the ground), while in the baseline condition, no taps were given. Participants' responses in the tactile leading or tactile lagging conditions were further recorded as either “congruent” or “incongruent.” For the tactile leading condition, responses were recorded as congruent if they were in the opposite of the direction of the initial tactile motion (a “left” response for initial rightward motion was recorded as congruent). In the tactile lagging condition, responses were recorded as congruent if they were in accordance with the direction of the initial tactile motion (a “left” response for initial leftward tactile motion was recorded as congruent), this recoding method was based on the perceived direction of tactile motion from the above different temporal structures and was in accordance with previous studies (Freeman and Driver, 2008; Chen et al., 2011).

The computer programs used in Experiments 1 and 2 were developed with Matlab (Mathworks Inc.) and the Psychophysics Toolbox (Brainard, 1997; Pelli, 1997). The test booth was semi-anechoic and dimly lit throughout the experiment, with ambient luminance of 0.05 cd/m^2^. The viewing distance was fixed at 60 cm, which was maintained by using a chin-rest.

#### Design and procedure

A 2 (posture: upright vs. inverted) × 4 (temporal structure: tactile leading, synchronous, lagging to the visual footstep, and baseline without taps) factorial design was adopted in this experiment. Participants were asked to report the perceived dominant walking direction of the point-light walker on the screen by pressing and holding the corresponding foot switch. The left switch was used to indicate leftward motion and the right switch was used to indicate rightward motion).

A complete cycle for the presentation of PLWs lasted 1300 ms. The total time duration for each single trial (i.e., the apparent motion of PLWs) was 70 s. Each condition was repeated and had five trials. The above tactile-visual temporal conditions were randomized and counterbalanced across all the trials. The inter-trial interval (ITI) between the two trials was 600–1000 ms. The onset of the first tactile stimulus was not started until 3000 ms (with a standard deviation of 500 ms) after the onset of the visual PLWs. The responses of the participants were not recorded for the first 10 s of each trial, beginning with the onset of the PLWs. This was done to prevent the initial bias of response arising from the first events (taps and visual PLWs), as shown in Figure 2.

**Figure 3:**
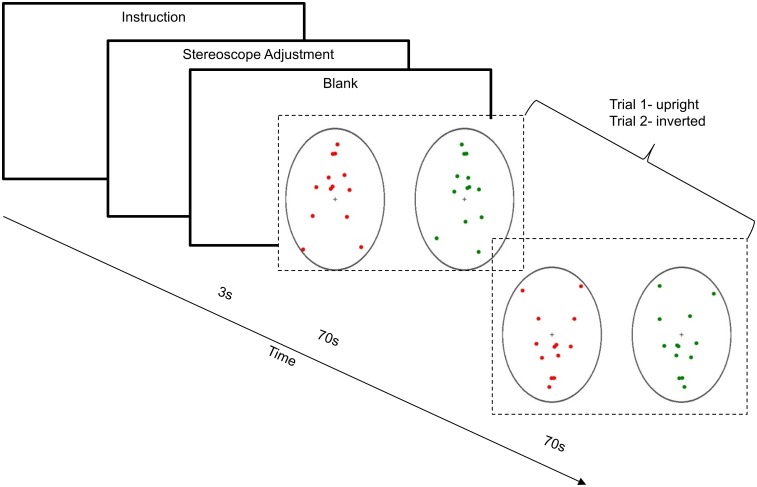
**Example trial for Experiment 1**. After the instructions and stereoscope adjustment, with a pause of 3 s, the trial started. During the 70 s cycle of the presentation of binocular PLWs, participants were required to hold down either the left foot-switch or right foot-switch to show the transition from dominant leftwards motion or dominant rightwards motion of the PLWs. This diagram shows the example of upright PLWs (trial 1) and inverted PLWs (trial 2).

Before taking part in the formal experiment, participants were asked to read the instructions and were provided with further detailed information related to the task when necessary. However, none of the participants knew the purpose of the experiment. The position of the stereoscope was adjusted in advance so that for each individual, the center of the point-light walkers could be perceived as overlapping before starting the experimental trials. A short video demonstration of the binocular PLWs was given before the formal experiment so that the participants would be familiar with the task. Then, they were trained in a pre-experiment with four trials containing each condition, to ensure they were capable of performing the required task. Each participant wore sponge earplugs and a headset to prevent any faint tactile noise during the experiment. During the experiment, they were required to focus on the central cross (fixation point) and report the perception of the dominant motion direction (leftwards vs. rightwards) of the perceived PLW projected through the stereoscope for 70 s by holding down the left foot-switch or right foot-switch, as shown in Figure 3. As explained earlier, the first 10 s of responses were not recorded.

After the formal experiment, we conducted a control test in which participants were asked to report the perceived dominant direction (leftwards or rightwards) of tactile apparent motion, based on the same temporal conditions as in the main experiment (tactile preceding 150 ms, synchronous, or lagging 150 ms to the visual footstep of one PLW). We examined whether different temporal intervals between taps give rise to the dominant directional perception of the tactile motion, as in Chen et al. (2011), which contribute to capturing the dominant directional perception of the PLWs.

#### Results

**Figure 4:**
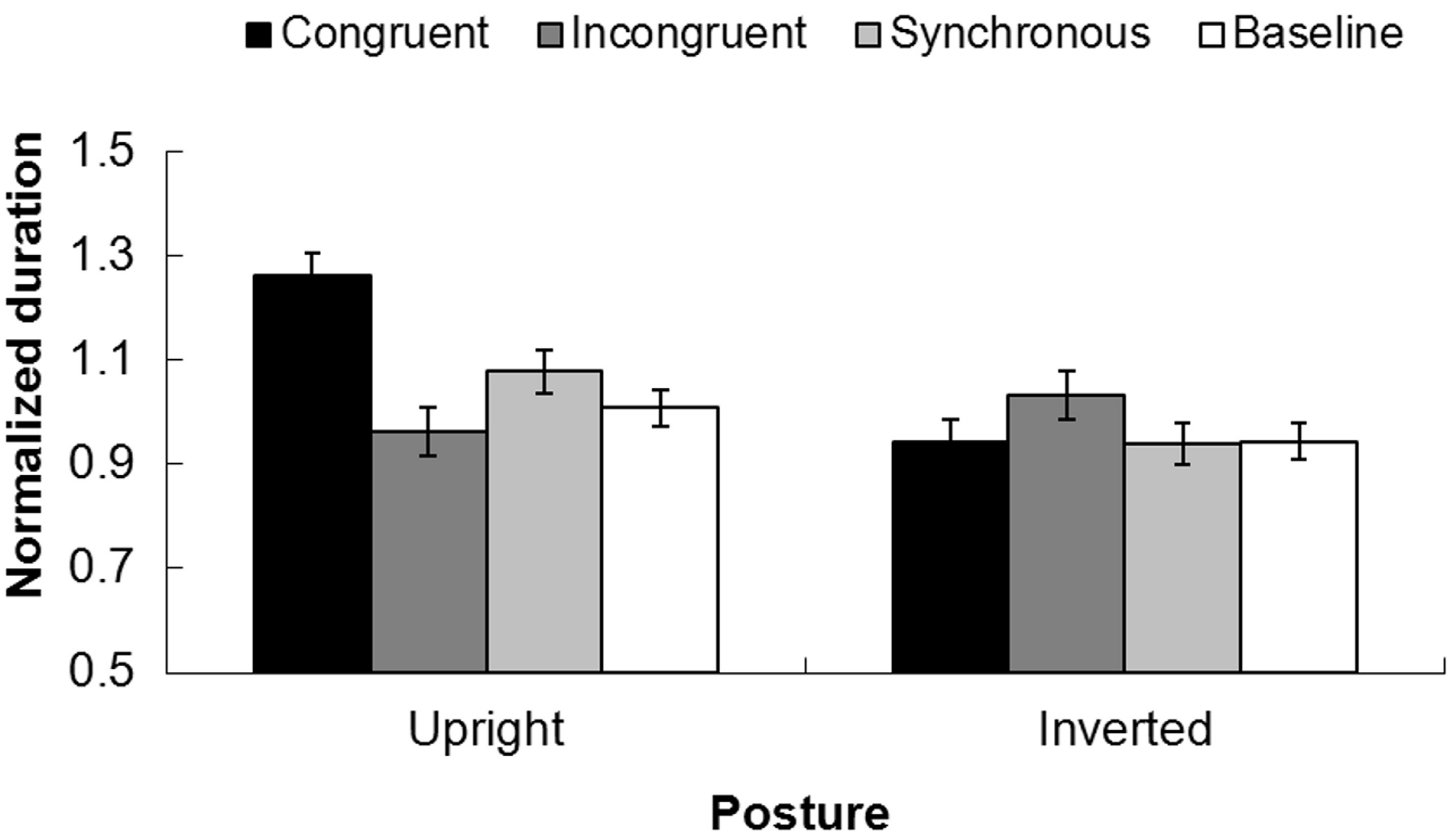
**Normalized durations for the perceived dominant direction of PLWs under different tactile-visual temporal structures with different postures (upright vs. inverted)**. The black column indicates the congruent condition, the dark gray column represents the incongruent condition, the light gray column shows the synchronous condition, and the white column shows the baseline. The error bars represent the standard errors of the mean.

The durations for holding the left switch or right switch were sorted separately by each temporal structure in upright and inverted postures. Since there was a large amount of individual variance, we normalized the duration by dividing the holding time with the mean across the four temporal conditions. The averaged normalized duration for all the participants are shown in Figure 4.

An Analysis of Variance (ANOVA) with the postures of point-light walkers (upright or inverted) and the recoded temporal conditions (“congruent,” “incongruent,” “synchronous,” and “baseline”) as independent factors and dominant durations as a dependent factor showed a significant main effect of posture, *F*_(1, 30)_ = 15.050, *p* < 0.01. The duration of the perceived normalized dominant direction for the upright point-light walker (Mean = 1.007, SEM = 0.185) was longer than the one in the inverted posture (Mean = 0.964 SEM = 0.174). The main effect for temporal conditions was also significant, *F*_(3, 90)_ = 3.558, *p* < 0.05. Bonferroni-corrected pairwise analysis showed the dominant duration in the congruent condition (Mean = 1.102, SEM = 0.225) was significantly longer than the ones in the synchronous condition (Mean = 1.008, SEM = 0.188) and baseline (Mean = 0.976, SEM = 0.169) conditions, but no difference between synchronous and baseline conditions, *p* > 0.05. The interaction between the temporal structure between tactile stimuli and visual stimuli and the posture was significant, *F*_(3, 90)_ = 7.645, *p* < 0.001.

A repeated measures ANOVA was implemented for upright and inverted postures separately. For the upright posture, normalized durations for congruent, incongruent, synchronous, and baseline conditions were 1.261 (0.044), 0.962 (0.047), 1.078 (0.041), and 1.008 (0.034), respectively. The main effect of the temporal structure was significant, *F*_(3, 45)_ = 14.448, *p* < 0.001. Bonferroni adjusted pairwise analysis showed the duration in the congruent condition (1.261) was significantly longer than the ones in the synchronous (1.078) and baseline (1.008) conditions, while the normalized duration in the incongruent condition (0.962) was significantly lower than the ones in the synchronous and baseline conditions, *p* < 0.05. For the inverted condition, the durations of the perceived dominant direction for congruent, incongruent, synchronous and baseline conditions were 0.942 (0.044), 1.033 (0.047), 0.939 (0.041), and 0.944 (0.034), respectively. In contrast to the results for the upright posture, however, the inputs for tactile stimuli imposed no noticeable influence upon the perceived dominant motion direction of PLWs, *F*_(3, 45)_ = 0.907, *p* = 0.436. This is shown in Figure 4).

In light of these results, it appears that the temporal structure of tactile stimuli resolved the ambiguity of perceived dominant direction information for the binocular PLWs. However, to obtain the modulation effect from the tactile feedback, the PLWs should take on upright postures, which resemble the normal stance for walking people and suggest ecological constraints during cross-modal influence. This will be addressed in more detail in the Discussion section.

**Figure 5:**
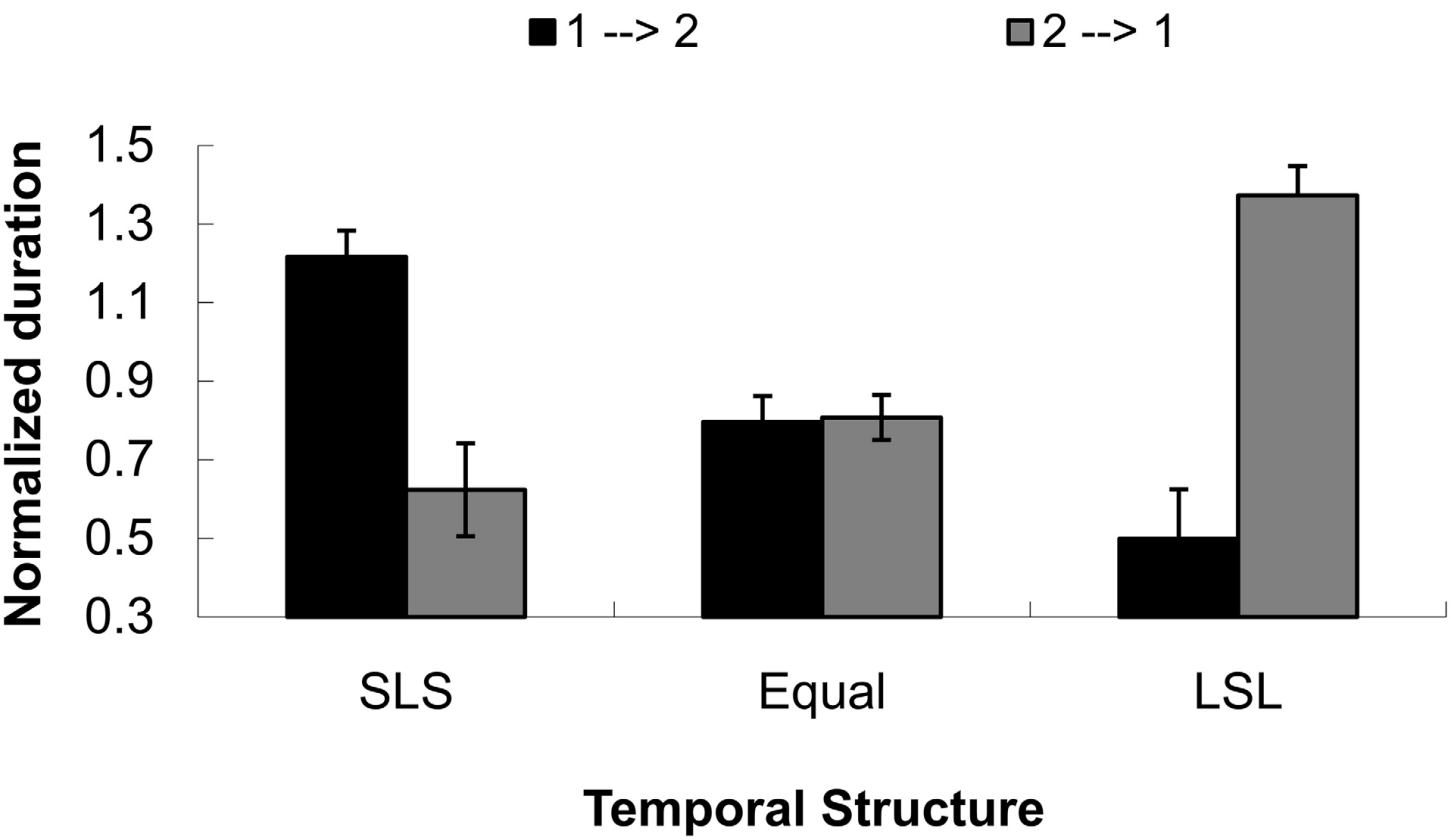
**Normalized duration for dominant directional perception in three temporal structures (short-long-short, equal interval and long-short) for a control test to Experiment 1**. The directions were defined as from the initial tap to the second tap (1→2) or from the second tap to the initial tap (2→1). SLS indicates the temporal structure of short-long-short, equal means equal temporal intervals, and LSL shows the temporal structure of long-short-long intervals.

Sixteen additional subjects from the same population (undergraduate students, 8 female, from Peking University, aged 18–23 years) participated in a control experiment to judge the dominant direction of tactile apparent motion in the absence of visual stimuli. The mean normalized duration for the direction that went from the initial tap to the second tap (i.e., 1→2) was 0.837(0.048), and for the direction that went from the second tap to the initial tap was 0.935(0.051). The main effect of direction was not significant, *F*_(1, 15)_ = 1.634, *p* = 0.221. The mean durations for SLS (short-long-short), equal (equal temporal intervals), and LSL (long-short-long) were 0.920(0.051), 0.802(0.032), and 0.936(0.042), respectively. The main effect of temporal condition was significant, *F*_(2, 30)_ = 4.336, *p* < 0.05. Bonferroni-corrected pairwise comparison showed the mean duration for the equal condition (0.802) was shorter than for the mean duration for LSL (0.936). Importantly, the interaction between direction and temporal condition was significant, *F*_(2, 30)_ = 19.418, *p* < 0.001. Further simple effects analysis with multivariate analysis of variance (MANOVA) indicated that the two perceived directions (1→2 and 2→1) were significantly different in the two SLS and LSL conditions, *F*_(1, 15)_ = 12.97, *p* < 0.01 and *F*_(1, 15)_ = 21.70, *p* < 0.001. However, there was no difference in the equal condition, *F* < 1, as shown in Figure 5.

The results indicated that the capture of visual apparent motion in PLWs could mainly be based on the information of the perceived dominant direction of tactile apparent motion, which captures the directional perception of PLWs.

## Experiment 2

The walking direction (leftwards vs. rightwards) in Experiment 1 as a means of horizontal movement is seldom observed in real life situations. Therefore, in Experiment 2, we adopted receding/approaching walking postures to simulate the more common daily walking style. In addition, in order to better simulate the natural somatosensory perception related to walking, we moved the tactile stimuli from the fingertips to the ankles. In Experiment 2 we were interested in how the social-cognitive factor of empathy modulates the cross-modal (tactile-visual) temporal dynamic capture of the perceived direction of PLWs.

### Method

#### Participants

Twenty-six undergraduates (ten female) from Peking University, aged 19–24 years, who met the same requirements of Experiment 1 participated in this experiment. The experiment was performed in compliance with all institutional guidelines set by the Academic Affairs Committee of the Department of Psychology at Peking University. All participants provided written informed consent according to institutional guidelines and the Declaration of Helsinki. Participants were reimbursed at a 20RMB/hour rate.

#### Stimuli, apparatus, and procedure

The same apparatus and tactile stimuli of Experiment 1 were used in Experiment 2, except that the tactile actuators were attached to the front *and* back side of the ankle area, rather than on the fingertips. Two taps were put on the back of the two ankles while another two vibrators were put on the front of the ankles. All the PLWs took upright postures.

For the tactile stimuli, four stimuli were presented, with two attached to each ankle, either on the front or the back side of it. Tactile stimuli on the same side (e.g., front) were always presented at the same time, but the time interval between front and back side taps was manipulated with the same temporal structures as in Experiment 1. The tactile stimuli used in this study could simply be seen as the tactile stimuli used in Experiment 1, but rotated horizontally to the vertical motion, by attaching the tactile stimuli to each of the ankles. Participants were informed that while they could perceive the directional information of the tactile stimuli, the taps were irrelevant for determining the directions (receding vs. approaching) of the PLWs.

**Figure 6:**
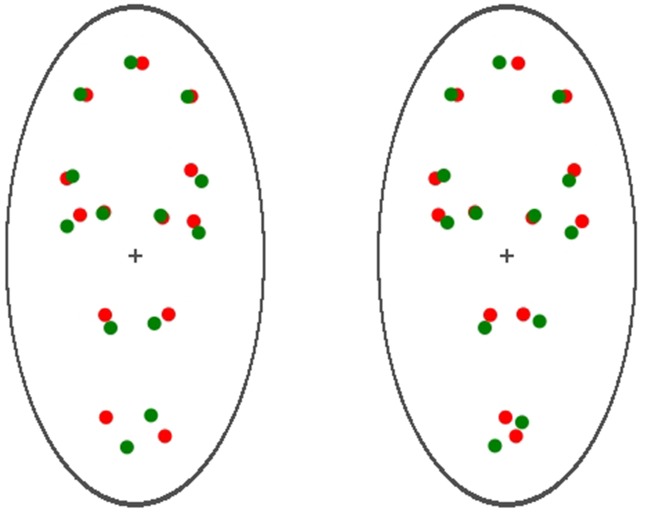
**Visual stimuli used in Experiment 2**. Two red and green PLWs were displayed on both the left and the right half of the screen. A minor angular rotation (7° disparity) relative to its vertical location was applied to each PLW, so that the walking direction of the PLW on the left visual field was 97°, while that of the PLW on the right visual field was 83° (in reference to the right-hand X-axis for both). Observed through a stereoscope, the walking direction of the PLW appeared as either facing away from (receding) or toward (approaching) the participants.

To render the binocular visual stimuli, two red and green PLWs were displayed on both the left and the right half of the screen and adjusted with a minor angular rotation (7° disparity) relative to its vertical location. Doing so ensured that the walking direction of the PLW on the left visual field was 97° while that of the PLW on the right visual field was 83° (in reference to the right-hand X-axis for both). Note that the walking direction of the PLW appeared either facing away from (receding) or toward (approaching) the participants, as shown in Figure 6. These settings guaranteed the ambiguous nature of the apparent motion for the PLWs, and that for the given time period (70 s, with the same recording method as in Experiment 1), the participants could report their subjective dominant perception of the PLWs: either receding from or approaching themselves. The data was recorded by pressing and holding down two buttons of a custom-made response box (interfaced with a parallel port of the computer).

Similarly, we would expect that the temporal organization of tactile motion *per se* contributes to the observed cross-modal dynamic capture effect. A baseline task was implemented after the experiment to examine the effect of the temporal structure of the tactile stimuli upon the perceived dominant direction (receding vs. approaching) of the tactile apparent motion.

After the behavioral experiment, we asked the participants to fill in the Interpersonal Reactivity Index scale (Chinese version, IRI-C) (Rong et al., 2010), which includes four sub-scales of perspective-taking (PT), fantasy (FS), empathic concern (EC), and personal distress (PD); see the IRI-C is presented in the Supplementary Material. Based on the scores and according to common practice as described in above literature, we separated the individuals into two groups: a higher empathy group (with higher scores) and a lower empathy group (with lower scores), according to the above the median and below the median value of the scores (IRI ≥ 39, high empathy group; and IRI ≤ 38, low empathy group; 38 was the median).

## Results

### Cross-modal temporal capture effect

The mean normalized durations for congruent, incongruent, synchronous, and baseline conditions were 1.402(0.076), 0.694(0.046), 0.942(0.049), and 1.067(0.038), respectively. A repeated measures ANOVA with temporal congruency as the independent variable showed a significant main effect of congruency, *F*_(3, 75)_ = 24.16, *p* < 0.001. Bonferroni-corrected pairwise analysis showed that the duration for the congruent condition (1.402) was longest (*p'*s < 0.01) and the duration for the incongruent condition (0.694) was shortest (*p'*s < 0.05) among the four temporal structures. However, the duration for the synchronous condition (0.942) was statistically equal to the one in the baseline condition (1.067), *p* > 0.05. This result pattern suggests a significant impact of the cross-modal temporal structures on the perceived dominance of directional information for PLWs, just as we observed in Experiment 1.

### Baseline tests: facing-the-viewer bias and perceived direction for tactile apparent motion

**Figure 7:**
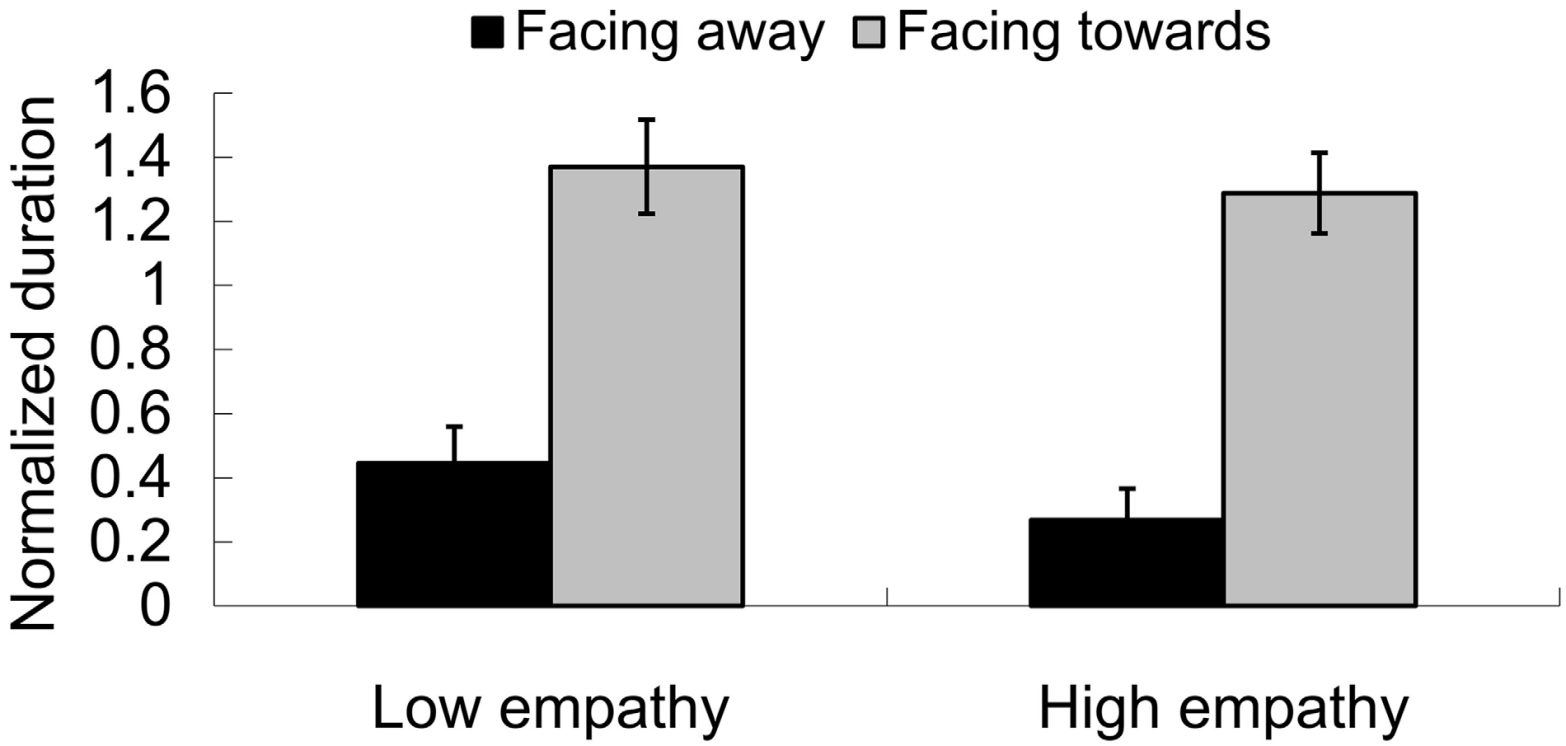
**Facing-the-viewer bias for PLWs**. In both the low empathy group and high empathy group, the proportion of reporting approaching (facing toward observers) was higher than the one of reporting receding (facing away from observers).

In the visual-only condition, the normalized duration for a receding perception (facing away from the observer) was 0.356 (0.076) and for an approaching perception (facing toward the observer) was 1.329 (0.097), *F*_(1, 24)_ = 54.539, *p* < 0.001. Therefore, a facing-the-viewer bias was manifested. This replicates several studies reported on in the literature (Vanrie et al., 2004; Brooks et al., 2008; Miller and Saygin, 2013; Van de Cruys et al., 2013; Heenan and Troje, 2014). However, there was no main effect of group. The mean duration for the low empathy group was 0.907 (0.086) and 0.778 (0.073), *F*_(1, 24)_ = 1.311, *p* = 0.264. Also, there was no interaction effect between group and direction, *F*_(1, 24)_ = 0.129, *p* = 0.722, as shown in Figure 7.

An additional control test (14 participants from Peking University, aged from 18 to 24 years old) discriminating the perceived direction of tactile apparent motion) showed that indeed, the temporal (interval) structure between tactile events caused a subjective bias of the perceived dominant direction of tactile apparent motion. The main effect of direction was not significant, *F*_(1, 13)_ = 3.476, *p* = 0.085. The main effect of temporal condition was also not significant, *F*_(2, 26)_ = 1.463, *p* = 0.250. The interaction between direction and temporal condition, however, was significant, *F*_(2, 26)_ = 13.952, *p* < 0.001.

**Figure 8:**
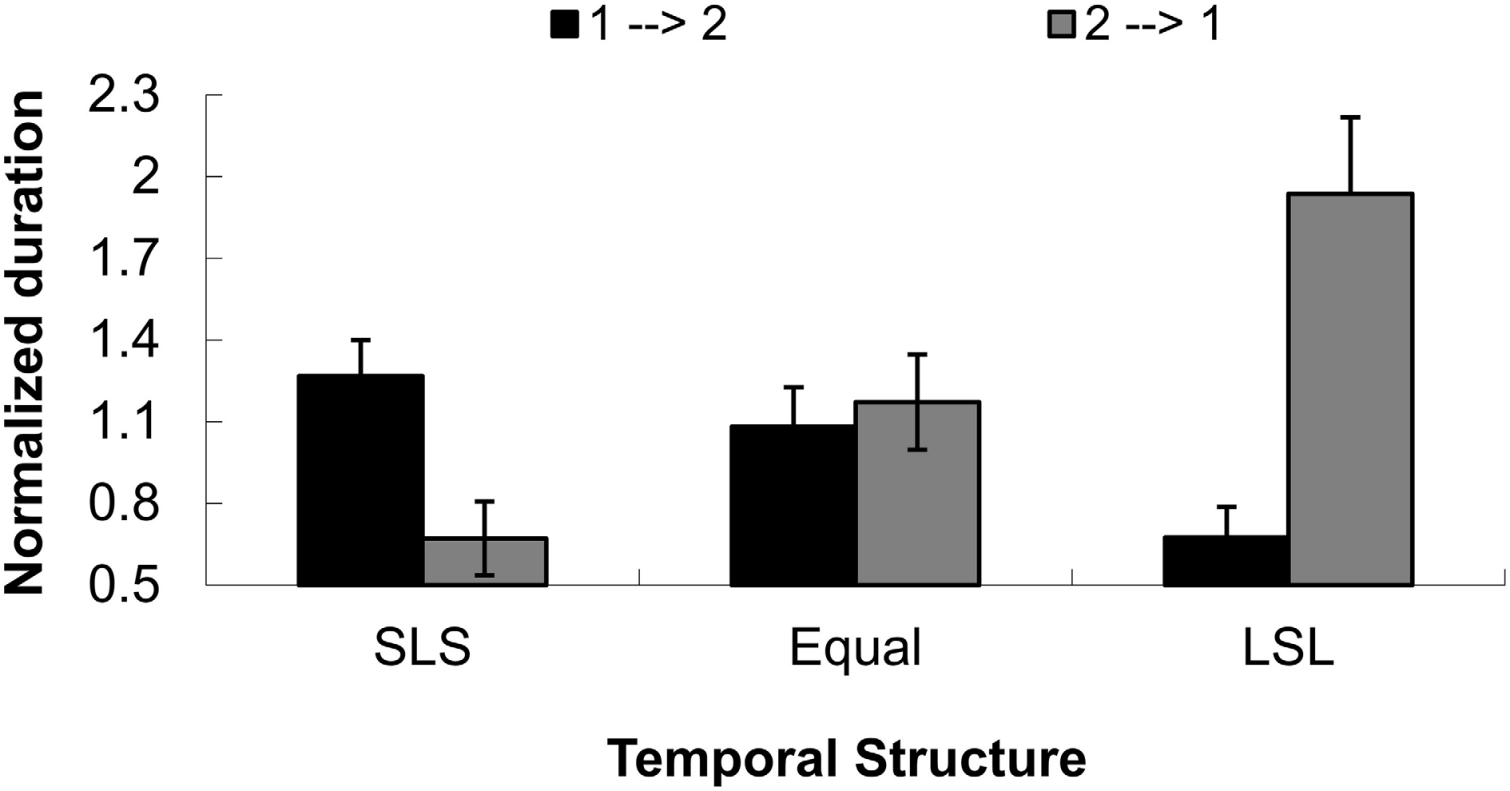
**Normalized duration for dominant directional perception in three temporal structures (short-long-short, equal interval and long-short) for the Experiment 2 control test**. The directions were defined as being from the initial tap to the second tap (1→2) or from the second tap to the initial tap (2→1). SLS indicates the temporal structure of short-long-short, Equal means equal temporal intervals, and LSL indicates the temporal structure of long-short-long intervals.

Further, simple effects analysis with MANOVA indicated that the two perceived directions (1→2 and 2→1) were significantly different in the two SLS and LSL conditions, *F*_(1, 13)_ = 7.23, *p* < 0.05 and *F*_(1, 13)_ = 18.19, *p* < 0.01, but not significantly different in the Equal condition, *F* < 1, as shown in Figure 8. This result pattern replicated the findings of the control test in Experiment 1, showing that the temporal structures between tactile events could lead to a dominant directional perception that gives rise to a capture effect in visual motion.

### The individual difference of high or low empathy

**Figure 9:**
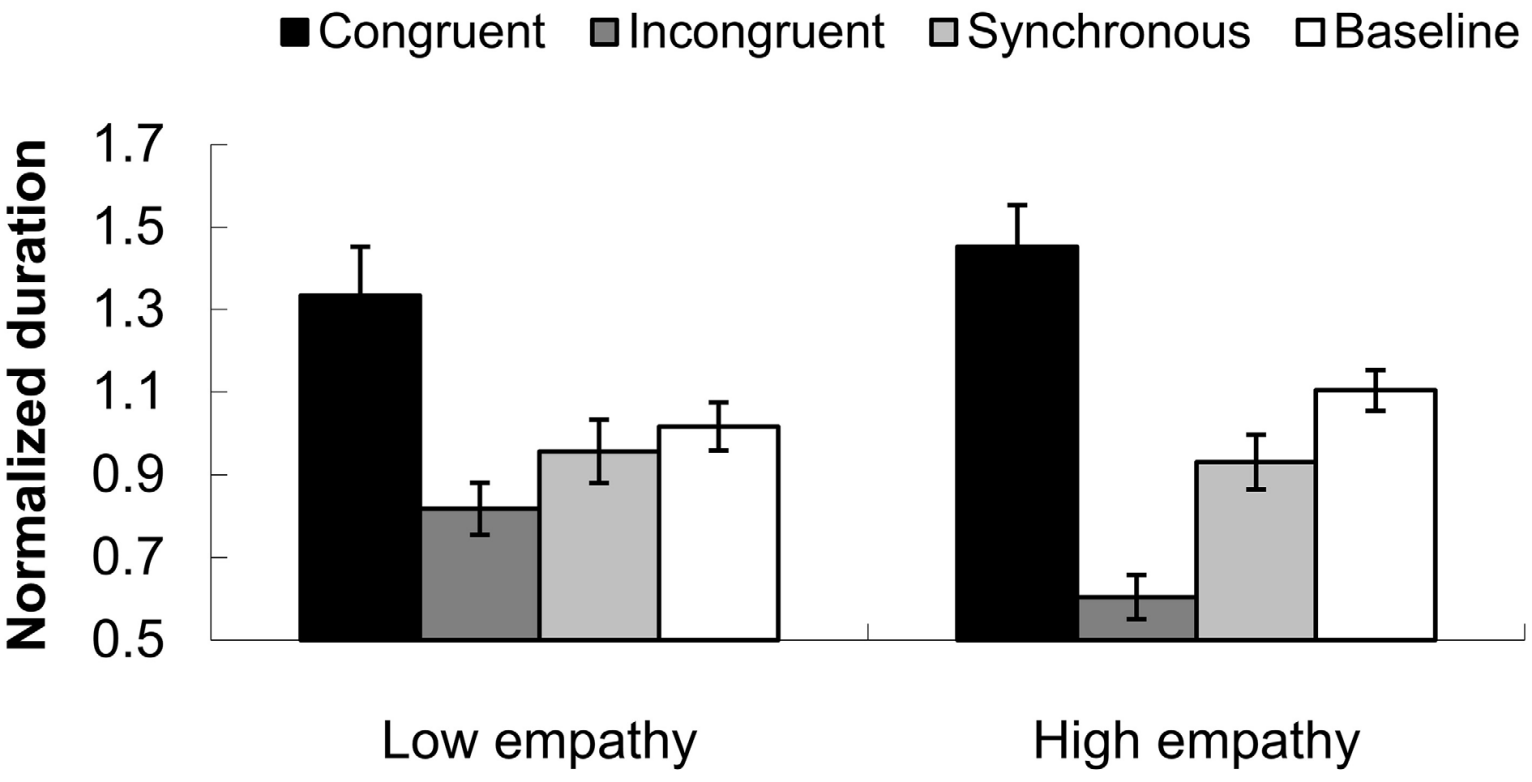
**Normalized durations for the perceived dominant direction of PLWs in lower and higher empathy groups**. The black column indicates the congruent condition, the dark gray column represents the incongruent condition, the light gray shows the synchronous condition, and white the baseline. The error bars represent standard errors of the mean.

We compared the performance of two groups (high empathy vs. low empathy). In the incongruent condition, a group difference was observed. Individuals with high empathy had a shorter normalized dominant duration 0.604 (0.054) than those with low empathy, with a mean duration of 0.818 (0.063), *F*_(1, 25)_ = 6.595, *p* < 0.05, as shown in Figure 9. This result pattern indicates that high empathy individuals were more readily captured by the tactile input. The tactile capture effect was shown mainly in the incongruent condition, in which the incongruent temporal structure between tactile events and biological motion somehow inhibited the perceived dominant directional information for PLWs.

The variances of the mean durations could also be used to measure the tactile capture effect on visual perception. The mean standard deviations for congruent, incongruent, synchronous, and baseline conditions were 1.143(0.071), 1.936(0.096), 1.550(0.067), and 1.608(0.062), respectively. The main effect of condition was significant, *F*_(3, 72)_ = 21.175, *p* < 0.001. Bonferroni-corrected pairwise comparisons showed that while there was no significant difference between synchronous (1.550) and baseline (1.608) conditions, the differences among the other cohorts were significant (*p*'s < 0.05). The group effect was not significant, *F*_(1, 24)_ = 0.004, *p* = 0.640. However, the interaction between temporal conditions and group was significant, *F*_(3, 72)_ = 21.175, *p* < 0.001. Further analysis using a One-Way ANOVA indicated that on the dimension of congruency, the variance was lower for the higher empathy group (1.014) than the variance for the lower empathy group (1.319), *F*_(1, 25)_ = 5.196, *p* < 0.05. This shows that for higher empathy individuals, the tactile capture effect was relatively stable in the congruency condition.

## Discussion and conclusion

In this study, we revealed that the perception of directional information for PLWs under binocular rivalry conditions could be resolved by using tactile inputs, which simulate the tactile feedback of visual footsteps hitting the ground. By systematically manipulating the temporal intervals between tactile and visual events, we first extended the cross-modal dynamic capture effect from the visual-auditory domain to the visual-tactile domain, using PLWs. Specifically, when the walking pace signaled by the tactile stimuli were temporally congruent with the visual PLWs, the temporal structure facilitated the dominant directional perception—either dominant leftwards/rightwards movement (Experiment 1) or dominant receding/approaching movement (Experiment 2), with increased normalized durations. However, when the temporal structure of tactile feedback was incongruent with the visual footsteps, the perceived dominant directional information was inhibited with reduced normalized durations. *Post-hoc* observations and control tests indicated that the observers had on chance level to report the temporal synchronies with 150 ms between the tactile stimuli and visual footsteps, suggesting that the temporal dynamic capture effect was largely genuine perceptual processing.

The capture effect was larger for the congruent condition, rather than the temporally synchronous condition. This result pattern was in agreement with some previous studies on cross-modal temporal dynamic capture (Freeman and Driver, 2008; Shi et al., 2010). The results for the control test of discerning the dominant direction of tactile apparent motion in the absence of visual events indicate that the cross-modal dynamic capture effect was mainly driven by the perceived directional information of tactile events. In the unisensory modality (the tactile modality), the variation in temporal intervals between tactile inputs caused a potent directional perception of tactile motion (leftwards/rightwards in Experiment 1, and facing toward/away in Experiment 2), which further captured the perceived dominant direction of the PLWs. During the visual-tactile interaction, the intra-modality perceptual grouping might precede the cross-modal (visual vs. tactile) binding process to produce the capture effect (Keetels et al., 2007; Cook and Van Valkenburg, 2009; Roseboom et al., 2013). The capture effect was not shown in the “synchronous” condition, which was seemingly contradictory to the findings that use other paradigm such as visual Ternus apparent motion (Shi et al., 2010). For example, in Shi et al. (2010) the two tones synchronously paired with two visual frames would change the observers' categorization of motion percept (more “group motion” vs. “element motion”). Those differential findings are probably due to the differential tasks involved in different research paradigms. The current study used directional information of long-range apparent motion for probe, the capture effect stems from the build-up of the perceived temporal structure based on the varied temporal intervals (Freeman and Driver, 2008; Chen et al., 2011), which is absent in the “synchronous” condition. Therefore, we did not observe, if any, noticeable cross-modal capture effect when visual and tactile events were synchronous.

The cross-modal capture effect was observed in the upright visual configurations rather than in the inverted configurations, suggesting that cross-modal temporal capture is orientation specific (Pavlova and Sokolov, 2000), and that the sociobiological meaning (normal upright posture) of the biological motion is very important for detecting PLWs (Watson et al., 2004). This ecological constraint of perceiving PLWs was also shown in other studies (Cutting et al., 1988; Mather et al., 1992; Bertenthal and Pinto, 1994; Neri et al., 1998; Thornton, 1998). Pavlova and Sokolov (2000) reported an abrupt improvement in recognition of point-light walkers when the orientation changed from inverted to upright. These researchers used masking and priming procedures to investigate how display orientation affects recovery of a known point-light figure and found a high sensitivity to a camouflaged point-light walker with an upright orientation. A priming effect in biological motion was observed only if a prime corresponded to a range of deviations from the upright orientation within which the display was spontaneously recognizable. In their masking and priming paradigms, the recovery of a coherent structure is connected primarily with top-down processing of biological motion. However, their results indicated that orientation influences bottom-up processing of biological motion and influences top-down processing less. In Experiment 1 of our study, ecological constraints in perceiving PLWs were also shown. Here, the cross-modal capture effect on PLWs was observed with the upright posture, but not with the inverted posture.

We further showed that the capture pattern was modulated by empathy. Generally, high empathy individuals were more readily influenced by tactile inputs, with the characteristic capture effect in the incongruent condition. That is, high empathy group showed decreased normalized duration in the incongruent condition, compared to the low empathy group. High empathy individuals also demonstrated relatively stable performance with small variance (standard deviations) for the normalized duration in the congruent condition. These results suggest that multisensory interaction can be modulated by an individual's cognitive traits, and conform to an unwritten social norm. This effect might arise in people with high anxiety, as mistaking an approaching person for someone who is receding might have more severe consequences than the opposite mistake (Van de Cruys et al., 2013; Weech et al., 2014). People with higher empathic concern might be more sensitive to the direction of conflicting sensory cues (as in the incongruent condition), so as to avoid a potential mistake, like those in the high-anxiety group just mentioned. With the enhanced shared (mirror) touch experience of the first-person (the participant) and the third person (the PLWs), people with higher empathic concern could better exploit the vicarious somatosensory responses for simple touch and be more sensitive to others' situations, including suffering (Banissy and Ward, 2007). In the current experimental scenario, the modulation arising from the factor of individual differences magnifies the difference of the temporal ventriloquism effect (tactile captures visual) between the high empathy group and low empathy group.

Other researchers have also recently found that individual differences in cognitive traits can influence the perception of PLWs. For example, with respect to ambiguous visual stimuli, more anxious individuals display a bias toward perceiving a more threatening image compared to those who are less anxious (Fox et al., 2002; Gray et al., 2009; Singer et al., 2012; Van de Cruys et al., 2013; Heenan and Troje, 2014). Heenan and Troje (2014) presented data to support that the facing-the-viewer bias is influenced at least in part by the social relevance of biological motion stimuli. Individuals with high anxiety level demonstrate a higher degree of facing-the-viewer bias than individuals with low anxiety. Evidence from the clinical field has shown that people with higher levels of Autism Spectrum Disorder have impaired global, but compensatory local, biological motion processing (van Boxtel and Lu, 2013). The studies cited have shown that personal cognitive/emotional states, whether in normally developing or atypically developing groups, could shape the perception of PLWs. Our study provides further evidence to support the idea that social-cognitive abilities can effectively modulate the otherwise ambiguous perception of point-light walkers. However, there might be individual differences in the ability to complete tasks that rely more heavily on the use of different cues in biological motion (form vs. motion and translational cues) (Rybarczyk and Santos, 2006; Wang et al., 2010; Miller and Saygin, 2013). Moreover, further study should aim to elucidate the intricate mechanisms underlying how individual differences modulate cross-modal interaction, as we have observed with the paradigm of PLWs.

Taken together, the above evidence suggests that tactile input helped to resolve the otherwise ambiguous perception of biological motion, and that this cross-modal effect is modulated by higher level social-cognitive factors, such as empathic concern.

### Conflict of interest statement

The authors declare that the research was conducted in the absence of any commercial or financial relationships that could be construed as a potential conflict of interest.
