# Corrigendum: Tactile input and empathy modulate the perception of ambiguous biological motion

**DOI:** 10.3389/fpsyg.2015.01384

**Published:** 2015-09-08

**Authors:** Hörmetjan Yiltiz, Lihan Chen

**Affiliations:** ^1^Department of Psychology, Peking UniversityBeijing, China; ^2^Key Laboratory of Machine Perception (Ministry of Education), Peking UniversityBeijing, China

**Keywords:** tactile, point-light walker, temporal, empathy, apparent motion, binocular rivalry

The citation of:

Weech, S., McAdam, M., and Troje, N. F. (2014). What causes the facing-the-viewer bias in biological motion? *J. Vis*. 14, 1–15. doi: 10.1167/14.12.10

should be corrected as:

Weech, S., McAdam, M., Kenny, S., and Troje, N. F. (2014). What causes the facing-the-viewer bias in biological motion? *J. Vis*. 14, 1–15. doi: 10.1167/14.12.10

The original article has been updated.

## Conflict of interest statement

The authors declare that the research was conducted in the absence of any commercial or financial relationships that could be construed as a potential conflict of interest.

