# Supplementary material for: Tactile input and empathy modulate the perception of ambiguous biological motion
Source: Front Psychol. 2015 Feb 20;6:161. doi: 10.3389/fpsyg.2015.00161 (PMC4335391; doi:10.3389/fpsyg.2015.00161)
Supplement: Supplementary file 1 [file DataSheet1.DOCX]

Supplementary Material

**INTERPERSONAL REACTIVITY INDEX**

The following statements inquire about your thoughts and feelings in a variety of situations. For each item, indicate how well it describes you by choosing the appropriate letter on the scale at the top of the page: A, B, C, D, or E. When you have decided on your answer, fill in the letter on the answer sheet next to the item number. READ EACH ITEM CAREFULLY BEFORE RESPONDING. Answer as honestly as you can. Thank you.

ANSWER SCALE:

A B C D E

DOES NOT DESCRIBES ME

DESCRIBE ME VERY

WELL WELL

1. I daydream and fantasize, with some regularity, about things that might happen to me. (FS)

2. I often have tender, concerned feelings for people less fortunate than me. (EC)

3. I sometimes find it difficult to see things from the "other guy's" point of view. (PT) (-)

4. Sometimes I don't feel very sorry for other people when they are having problems. (EC) (-)

5. I really get involved with the feelings of the characters in a novel. (FS)

6. In emergency situations, I feel apprehensive and ill-at-ease. (PD)

1. I am usually objective when I watch a movie or play, and I don't often get completely caught up in it. (FS) (-)
2. I try to look at everybody's side of a disagreement before I make a decision. (PT)
3. When I see someone being taken advantage of, I feel kind of protective towards them. (EC)
4. I sometimes feel helpless when I am in the middle of a very emotional situation. (PD)

11. I sometimes try to understand my friends better by imagining how things look from their perspective. (PT)

12. Becoming extremely involved in a good book or movie is somewhat rare for me. (FS) (-)

13. When I see someone get hurt, I tend to remain calm. (PD) (-)

14. Other people's misfortunes do not usually disturb me a great deal. (EC) (-)

1. If I'm sure I'm right about something, I don't waste much time listening to other people's

arguments. (PT) (-)

16. After seeing a play or movie, I have felt as though I were one of the characters. (FS)

17. Being in a tense emotional situation scares me. (PD)

1. When I see someone being treated unfairly, I sometimes don't feel very much pity for them.

(EC) (-)

1. I am usually pretty effective in dealing with emergencies. (PD) (-)

20. I am often quite touched by things that I see happen. (EC)

1. I believe that there are two sides to every question and try to look at them both. (PT)
2. I would describe myself as a pretty soft-hearted person. (EC)

23. When I watch a good movie, I can very easily put myself in the place of a leading

character. (FS)

24. I tend to lose control during emergencies. (PD)

1. When I'm upset at someone, I usually try to "put myself in his shoes" for a while. (PT)
2. When I am reading an interesting story or novel, I imagine how I would feel if the events in the story were happening to me. (FS)

27. When I see someone who badly needs help in an emergency, I go to pieces. (PD)

28. Before criticizing somebody, I try to imagine how I would feel if I were in their place. (PT)
